# Racial disparity in tumor microenvironment and distant recurrence in residual breast cancer after neoadjuvant chemotherapy

**DOI:** 10.1038/s41523-023-00547-w

**Published:** 2023-06-13

**Authors:** Gina Kim, Burcu Karadal-Ferrena, Jiyue Qin, Ved P. Sharma, Isabelle S. Oktay, Yu Lin, Xianjun Ye, Saeed Asiry, Jessica M. Pastoriza, Esther Cheng, Nurfiza Ladak, John S. Condeelis, Esther Adler, Paula S. Ginter, Timothy D’Alfonso, David Entenberg, Xiaonan Xue, Joseph A. Sparano, Maja H. Oktay

**Affiliations:** 1 Department of Surgery, Albert Einstein College of Medicine/Montefiore Medical Center, Bronx, NY USA; 2 Department of Pathology, Albert Einstein College of Medicine/Montefiore Medical Center, Bronx, NY USA; 3 Department of Basic Oncology, Hacettepe University, Ankara, Turkey; 4 Department of Epidemiology & Population Health, Albert Einstein College of Medicine/Montefiore Medical Center, Bronx, NY USA; 5 Bio-Imaging Resource Center, The Rockefeller University, New York, NY USA; 6 College of Art and Sciences, New York University, New York, NY USA; 7 Gruss-Lipper Biophotonics Center, Albert Einstein College of Medicine/Montefiore Medical Center, Bronx, NY USA; 8 Integrated Imaging Program, Albert Einstein College of Medicine/Montefiore Medical Center, Bronx, NY USA; 9 Department of Pathology, Batterjee Medical College, Jeddah, Saudi Arabia; 10 CPL Pathology, Austin, TX USA; 11 Department of Pathology, NYU Grossman School of Medicine, New York, NY USA; 12 Department of Cell Biology, Albert Einstein College of Medicine/Montefiore Medical Center, Bronx, NY USA; 13 Department of Pathology, NYU Long Island School of Medicine, Mineola, NY USA; 14 Department of Pathology, Memorial Sloan Kettering Cancer Center, New York, NY USA; 15 Division of Hematology/Oncology, Icahn School of Medicine at Mount Sinai, Tisch Cancer Institute, New York, NY USA

**Keywords:** Breast cancer, Cancer microenvironment, Metastasis

## Abstract

Black, compared to white, women with residual estrogen receptor-positive (ER+) breast cancer after neoadjuvant chemotherapy (NAC) have worse distant recurrence-free survival (DRFS). Such racial disparity may be due to difference in density of portals for systemic cancer cell dissemination, called TMEM doorways, and pro-metastatic tumor microenvironment (TME). Here, we evaluate residual cancer specimens after NAC from 96 Black and 87 white women. TMEM doorways are visualized by triple immunohistochemistry, and cancer stem cells by immunofluorescence for SOX9. The correlation between TMEM doorway score and pro-metastatic TME parameters with DRFS is examined using log-rank and multivariate Cox regression. Black, compared to white, patients are more likely to develop distant recurrence (49% vs 34.5%, *p* = 0.07), receive mastectomy (69.8% vs 54%, *p* = 0.04), and have higher grade tumors (*p* = 0.002). Tumors from Black patients have higher TMEM doorway and macrophages density overall (*p* = 0.002; *p* = 0.002, respectively) and in the ER+/HER2- (*p* = 0.02; *p* = 0.02, respectively), but not in the triple negative disease. Furthermore, high TMEM doorway score is associated with worse DRFS. TMEM doorway score is an independent prognostic factor in the entire study population (HR, 2.02; 95%CI, 1.18–3.46; *p* = 0.01), with a strong trend in ER+/HER2- disease (HR, 2.38; 95%CI, 0.96–5.95; *p* = 0.06). SOX9 expression is not associated with racial disparity in TME or outcome. In conclusion, higher TMEM doorway density in residual breast cancer after NAC is associated with higher distant recurrence risk, and Black patients are associated with higher TMEM doorway density, suggesting that TMEM doorway density may contribute to racial disparities in breast cancer.

## Introduction

Breast cancer is the most common cancer and the second leading cause of cancer-related deaths among women^1^. Although breast cancer related deaths declined in the last three decades by approximately 40%, death rates declined less in Black, compared to non-Black, patients, resulting in persistent racial disparities in survival after a breast cancer diagnosis. Factors contributing to the racial gap in breast cancer mortality include: (i) advanced stage at presentation, (ii) higher incidence of triple-negative breast cancer (TNBC), (iii) higher comorbidity rates, (iv) poorer adherence to chemotherapy and endocrine therapy, and (v) adverse social determinants of health contributing to limitations in access to care^2–6^. Racial disparities are also present in clinical trial populations that are healthier and have access to care, indicating that social determinants of health and presentation with more advanced stage disease are the contributing factors^7–10^, and suggesting that other unknown factors may be contributing.

Due to more aggressive and advanced disease at presentation, Black patients with breast cancer are frequently treated with neoadjuvant chemotherapy (NAC) to downsize tumors and obtain information regarding treatment response. Tumor response to NAC is used as a pharmacodynamic biomarker that provides prognostic information and may be used to guide the choice of subsequent systemic adjuvant therapy after surgery^11,12^.

After NAC, the tumor microenvironment (TME) enters a reparative stage that results in a “cytokine storm” causing recruitment of bone marrow derived progenitors including pro-angiogenic TIE2+ monocytes and endothelial progenitor cells into TME^13,14^. In murine mammary carcinoma models, NAC leads to an increased density of tumor microenvironment of metastasis (TMEM) doorways, TMEM doorway-mediated cancer cell intravasation, and, ultimately, metastatic burden^15,16^. TMEM doorways are three-cell structures (composed of an actin regulatory protein mammalian-enabled [Mena] expressing tumor cell, a perivascular macrophage [TIE2 high], and an endothelial cell) and function as portals that surrounding tumor cells can use to intravasate into the blood stream^17–22^. Several large studies showed that TMEM doorway density is an independent prognostic marker for distant recurrence in patients with ER+/HER2- breast cancer who were treated with adjuvant systemic therapies^20,22,23^. It has been recently shown that TMEM doorways are also microanatomical niches enriched for cancer stem cells (CSCs) expressing SOX9^24^. This finding is important because CSCs have the ability to initiate primary tumor growth and metastatic foci at distant sites^25^. However, the importance of CSC density for disease outcome is inconclusive, as several studies have found an association between CSCs and inferior clinical outcome in breast cancer patients^26,27^, whereas others have not^28^. Moreover, in ER+/HER2- disease, TMEM doorway density increased in the residual disease after NAC when compared with TMEM doorway density in the primary tumor before NAC^15^. This effect is now recognized as a mechanism of resistance to cytotoxic therapy wherein tumor dissemination is promoted, despite concomitant cytoreduction^29^. In a pooled analysis of 9702 women from 8 National Surgical Adjuvant Breast and Bowel Project (NSABP) trials treated with NAC, Black patients with residual ER+ breast cancer after NAC had inferior DRFS compared to white patients^30^.

We hypothesize that racial differences in TMEM doorway density may contribute to a racial difference in clinical outcome. Here we test this hypothesis using a prospective-retrospective study of TMEM doorway density and TMEM doorway-associated TME after NAC.

## Results

### Patient characteristics

**Fig. 1:**
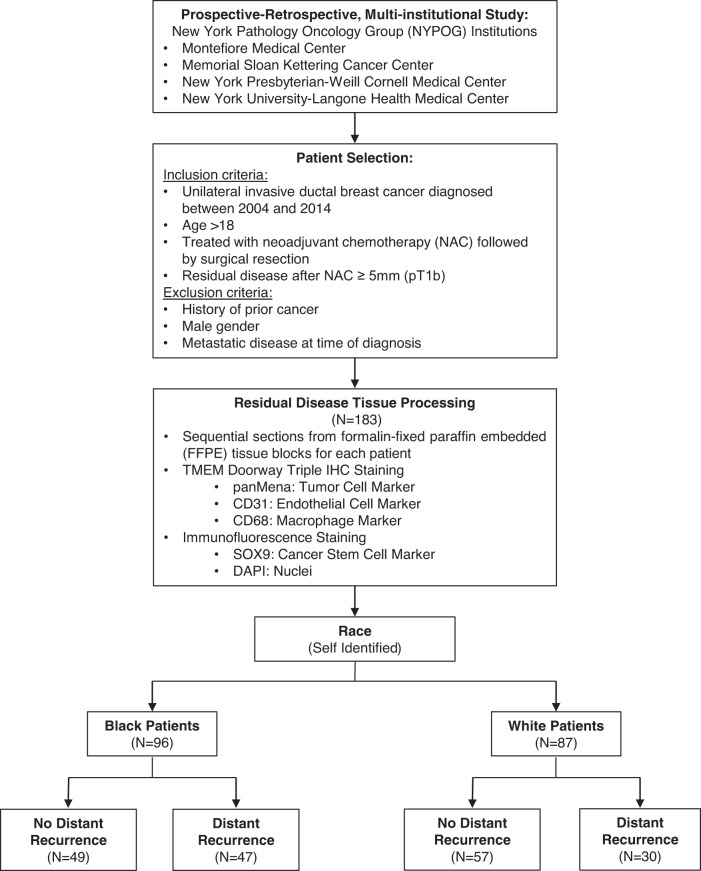
CONSORT diagram of the study.

We analyzed formalin-fixed paraffin-embedded tissue samples from 183 patients with residual invasive ductal breast cancer after NAC (Fig. 1). Tissue and outcome data from 96 (52.5%) Black and 87 (47.5%) white patients were included in the analysis (Table 1). There was no difference in neoadjuvant chemotherapy regimens (taxane only, taxane-containing, no-taxane containing regimens) between Black and white patients. Additionally, NAC only and NAC in combination with endocrine, HER2 inhibition, or radiation therapies were all similar in Black compared to white patients (Supplementary Table 1). When the cohort was stratified by breast cancer subtype, treatment regimens in Black and white patients with ER+/HER2- and TN disease were similar (Supplementary Table 2).

**Table 1:** Patient characteristics, entire cohort.

	Number of Patients (%)			
	All Patients *N* = 183 (100)	Black Patients *N* = 96 (52.5)	White Patients *N* = 87 (47.5)	*P*-Value
Distant Recurrence				0.07
Yes	77 (42.1)	47 (49)	30 (34.5)	
No	106 (57.9)	49 (51)	57 (65.5)	
Age				0.69
Mean [SD]	51.9 [11.5]	51.6 [10.3]	52.3 [12.7]	
Median [IQR]	52 [44, 60]	51 [45.8, 58.2]	52 [43.5, 61]	
Range	28–95	29–78	28–95	
Time to Distant Recurrence, Months				0.62
Mean [SD]	61.9 [40.4]	63.9 [42.9]	59.8 [37.7]	
Median [IQR]	62.5 [26.1, 92.9]	60.7 [26.6, 94.3]	64 [25.8, 89.5]	
Range	1–160.7	9.4–159.8	1–160.7	
Surgery				0.04
Mastectomy	114 (62.3)	67 (69.8)	47 (54)	
Breast-Conserving Therapy	69 (37.7)	29 (30.2)	40 (46)	
Tumor Stage (ypT)				0.84
T1 ( < 2 cm)	82 (44.8)	45 (46.9)	37 (42.5)	
T2 (2–5 cm)	71 (38.8)	36 (37.5)	35 (40.2)	
T3 ( > 5 cm)	30 (16.4)	15 (15.6)	15 (17.2)	
Lymph Node Status (ypN)				0.67
Positive	130 (71)	70 (72.9)	60 (69)	
Negative	53 (29)	26 (27.1)	27 (31)	
Grade				0.002
1	6 (3.3)	0 (0)	6 (6.9)	
2	50 (27.3)	22 (22.9)	28 (32.2)	
3	119 (65)	73 (76)	46 (52.9)	
Unknown	8 (4.4)	1 (1)	7 (8)	
Subtype				0.1
ER+/HER2-	91 (49.7)	41 (42.7)	50 (57.5)	
TNBC	59 (32.2)	37 (38.5)	22 (25.3)	
Other	33 (18)	18 (18.8)	15 (17.2)	

Wilcoxon rank sum test is used for continuous variables. Chi-squared tests or Fisher’s exact tests are used for categorical variables.

*SD* Standard deviation, *IQR* Interquartile range, *yPT* Tumor stage after neoadjuvant chemotherapy, *yPN* Lymph node status after neoadjuvant chemotherapy, *ER*+ Estrogen receptor positive, *TNBC* Triple negative breast cancer.

Demographic and tumor characteristics of the entire cohort, and for each breast cancer subtype, are described in Table 1 and Supplementary Table 3 respectively. Black, compared to white, patients were more likely to have distant recurrence (49% vs 34.5%, *p* = 0.07), receive mastectomy over breast conserving therapy (69.8% vs 54%, *p* = 0.04), and have a higher tumor grade (*p* = 0.002) in the entire cohort (Table 1). Age was similar among Black and white patients (51.6 vs 52.3). There was no difference in time to distant recurrence, tumor stage, lymph node status, or subtype between Black and white patients (Table 1).

When the cohort was stratified by breast cancer subtype, a racial difference in distant recurrence remained in ER+/HER2- (46.3% vs 28%) but did not reach statistical significance. However, this difference was lost in the TNBC (54.1% vs 54.5%) subtype (Supplementary Table 3). Black, compared with white, patients were more likely to get mastectomy (82.9% vs 52%, *p* = 0.004), have higher grade (*p* = 0.01), and positive lymph nodes (90.2% vs 66%, *p* = 0.01) in the ER+/HER2- subtype, but not in the TNBC subtype, indicating a racial disparity in the biology of ER+/HER2- but not TNBC disease. Black, compared to white, patients with ER+/HER2- breast cancer were slightly younger (49.6 vs 54), and patients with TNBC disease were slightly older (53.4 vs 50.9). Furthermore, there was no racial difference observed in the two breast cancer subtypes in time to distant recurrence and tumor stage (Supplementary Table 3).

### Approach to evaluating Pro-metastatic tumor microenvironment and distant recurrence

Since NAC may increase the density of TMEM doorways in certain patients, we wanted to determine if there is racial disparity in this recently reported side-effect of NAC^15,16,29^. In addition, we wanted to determine if TMEM doorway score could be used as prognostic indicator of metastasis in the residual disease post-NAC similarly to treatment-naïve breast cancers^20,22,23^. Given that macrophages and blood vessels are components of TMEM doorways we also investigated racial disparity in macrophage and microvascular density and potential correlation with DRFS of these 2 parameters of TME. Lastly, we wanted to determine racial disparity in CSC density because CSCs are crucial for initiation of growth of metastatic foci and it was demonstrated that TMEM doorways may act as niches where CSCs preferentially reside^24^.

In all analyses we first examined potential disparity in pro-metastatic parameters between the two most prevalent breast cancer subtypes, ER+/HER2- and TNBC, and then the racial disparity between Black and white patients.

### Disparity in pro-metastatic TME parameters

**Fig. 2:**
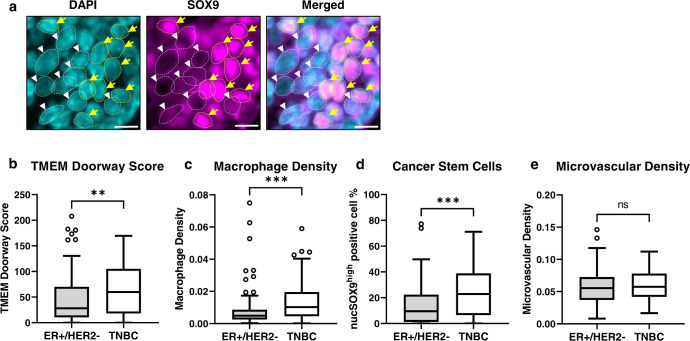
TME of TNBC, compared to ER+/HER2- breast cancer, is enriched for pro-metastatic markers, except for microvascular density. **a** Representative images for SOX9-high cells (yellow arrows) and -low cells (white arrowheads). Scale bar, 10 µm. **b** TMEM doorway score (*p* = 0.004). **c** Macrophage density (*p* = 0.0002). **d** Cancer stem cells (*p* = 0.0002). **e** Microvascular density (*p* = 0.44) levels in patients with ER+/HER2- breast cancer (*n* = 91) vs TNBC (*n* = 59). ER+ Estrogen receptor positive, TNBC Triple negative breast cancer, TME Tumor microenvironment. Mann-Whitney U-test is used for comparison between two-independent groups and box and whisker diagram is used for plotting the data (**b**–**e**). Box represents median value, lower and upper quartile values. Whiskers show minimum and maximum data values. Outliers are single data points that are more than 1.5 times of upper or lower quartiles. ns: not statistically significant, *p* > 0.05, *p ≤ 0.05, **p ≤ 0.01, ***p ≤ 0.001.

We used high nuclear expression of SOX9 to identify cells that activated stem program as previously reported^24^. Representative images of cancer cells expressing high levels of nuclear SOX9 (SOX9^high^) and low levels of nuclear SOX9 (SOX9^low^) are showed in Fig. 2a and representative images for TMEM doorway high vs mid/low analysis are shown in Supplementary Fig. 1. Tumors from patients with TNBC had a higher density of TMEM doorways (*p* = 0.004) (Fig. 2b), macrophages (*p* = 0.0002) (Fig. 2c), and CSCs (nuclear SOX9^high^ cancer cells, *p* = 0.0002) (Fig. 2d) than tumors from patients with ER+/HER2- breast cancer. There was no difference in microvascular density between these two subtypes of breast cancer (Fig. 2e). Of note, two cases were excluded from the analysis of microvascular density because of large amount of highly vascularized intertumoral stroma.

**Fig. 3:**
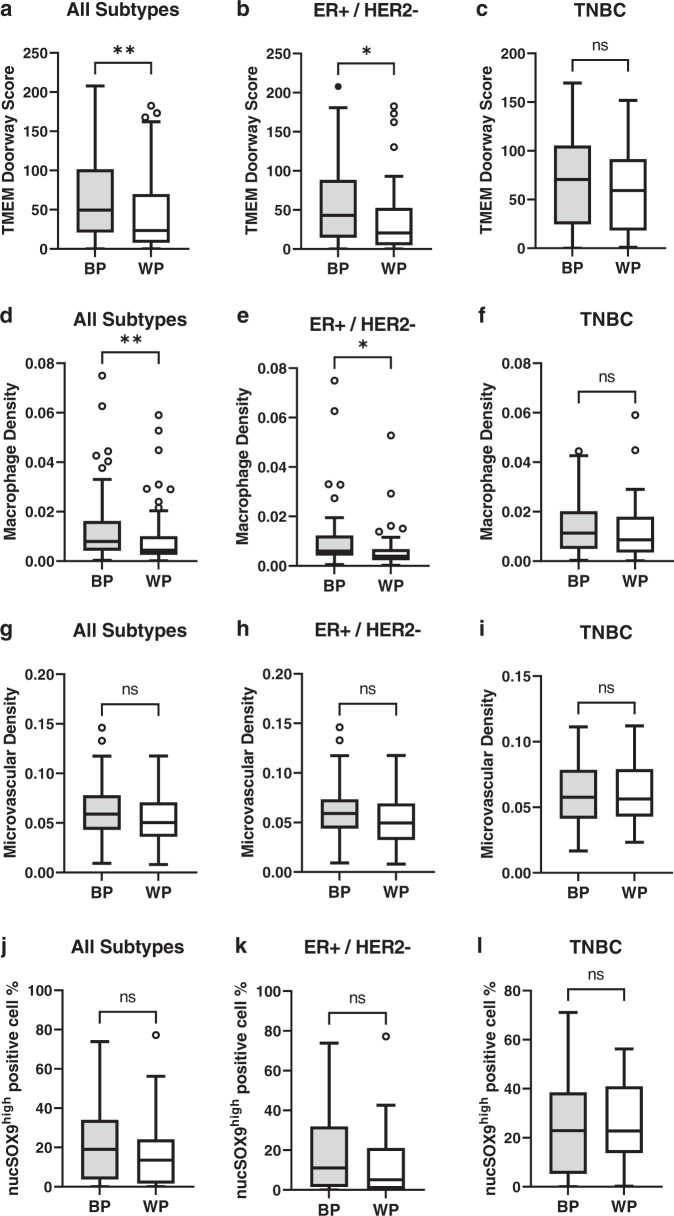
TMEM doorway score and macrophage density show racial disparity in the entire cohort and ER+HER2- breast cancer, but not in TNBC. **a**–**c** TMEM doorway score by race in all subtypes (**a**, *p* = 0.002), ER+/HER2- (**b**, *p* = 0.02), and TNBC (**c**, *p* = 0.74). **d**–**f** Macrophage density by race in all subtypes (**d**, *p* = 0.002), ER+/HER2- (**e**, *p* = 0.02), and TNBC (**f**, *p* = 0.31). **g**–**i** Microvascular density by race in all subtypes (**g**, *p* = 0.06), ER+/HER2- (**h**, *p* = 0.09), and TNBC (**i**, *p* = 0.97). **j**–**l** Cancer stem cell percentage in all subtypes (**j**, *p* = 0.09), ER+/HER2- (**k**, *p* = 0.09), and TNBC (**l**, *p* = 0.73). Patient numbers as follows: all subtypes BP (*n* = 96), WP (*n* = 87); ER+/HER2- BP (*n* = 41), WP (*n* = 50); TNBC BP (*n* = 37), WP (*n* = 22). BP Black patients, WP White patients, ER+ Estrogen receptor positive, TNBC Triple negative breast cancer. Mann-Whitney U-test is used for comparison between two-independent groups and box and whisker diagram is used for plotting the data (**a**–**l**). Box represents median value, lower and upper quartile values. Whiskers show minimum and maximum data values. Outliers are single data points that are more than 1.5 times of upper or lower quartiles. ns: not statistically significant, *p* > 0.05, *p ≤ 0.05, **p ≤ 0.01.

Black, compared to white, patients had higher density of TMEM doorways and macrophages in their TME overall (*p* = 0.002 and *p* = 0.002, respectively) and in ER+/HER2- subtype (*p* = 0.02 and *p* = 0.02, respectively) but not in TNBC (*p* = 0.74 and *p* = 0.31, respectively) (Fig. 3a–f). There was no racial difference in microvascular density (Fig. 3g–i) and density of CSCs (Fig. 3j–l) in either of the subtypes.

Taken together, these data show enrichment of the pro-metastatic TME parameters TMEM doorway and macrophage density in TNBC compared to ER+/HER2-. Additionally, when separated by race and subtype, Black, compared to white, patients had higher TMEM doorway and macrophage density only in ER+/HER2- but not in TN subtype. We next tested how high density of these pro-metastatic TME parameters correlate with DRFS.

### Correlation between DRFS and pro-metastatic TME parameters

Given that high TMEM doorway density is prognostic for DRFS in breast cancer patients receiving AC, we hypothesized that TMEM doorway density may also be prognostic for DRFS in patients with residual disease after NAC. We also wanted to examine if other pro-metastatic parameters in the residual disease post-NAC are associated with DRFS. All comparisons were made in the overall cohort as well as within ER+/HER2- and TNBC subtypes.

Since TMEM doorways contain macrophages and blood vessels, and since the areas around TMEM doorways have been found to be enriched for SOX9 expressing CSCs^24^, we first performed a Spearman correlation analysis among these pro-metastatic parameters and found significant positive correlation only between TMEM doorway score and macrophage density (Spearman Correlation Coefficient: 0.67) (Supplementary Fig. 2).

**Fig. 4:**
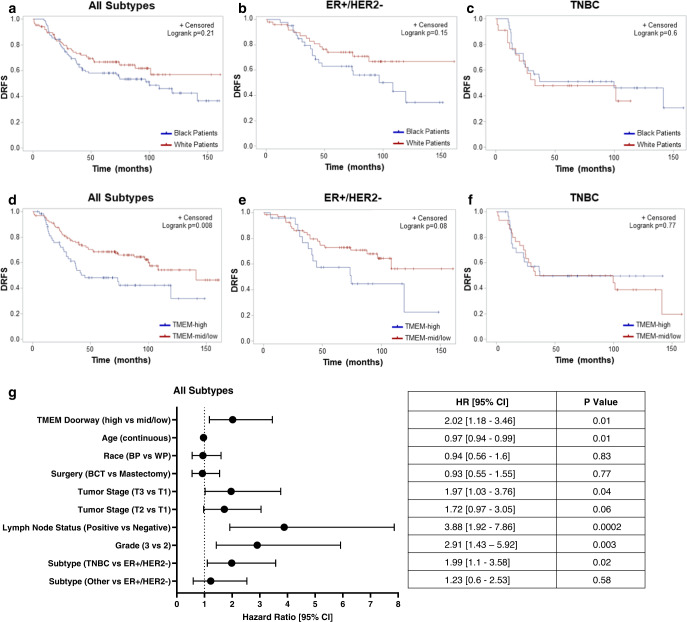
High TMEM doorway score is associated with inferior DRFS in the entire cohort. **a**–**c** DRFS in BP vs WP in the entire cohort (**a**, *p* = 0.21), ER+/HER2- breast cancer (**b**, *p* = 0.15), and TNBC (**c**, *p* = 0.6). **d**–**f** DRFS in TMEM-high vs TMEM-mid/low in patients in the entire cohort (**d**, *p* = 0.008), ER+/HER2- breast cancer (**e**, *p* = 0.08), and TNBC (**f**, *p* = 0.77). Kaplan-Meier survival curves and log-rank tests are used for DRFS analysis (**a**–**f**). **g** Cox regression model for covariates in the entire cohort (*n* = 175, patients with unknown status, *n* = 8, are excluded). Error bars represent 95% confidence intervals. Two-sided *p*-values are reported. DRFS: distant recurrence free survival, ER+ Estrogen receptor positive, TNBC Triple negative breast cancer, nucSOX9 Nuclear SOX9, BCT Breast conserving therapy, BP Black patients, WP White patients, HR Hazard Ratio, CI Confidence Interval.

Next, we examined the racial disparity in DRFS and observed a trend towards inferior DRFS for Black compared to white patients with ER+/HER2- disease, but not with TNBC (Fig. 4a–c). Even though we collected patient samples from multiple institutions, follow-up time was similar between Black and white patients (median follow-up time 68.95 and 71.53 months, respectively, *p* = 0.9). Then we studied the correlation of DRFS and pro-metastatic TME parameters. We found that patients with high TMEM doorway score, compared to patients with mid/low TMEM doorway score, in their residual disease had worse DRFS overall (*p* = 0.008) (Fig. 4d) and trended towards inferior DRFS in ER+/HER2- (*p* = 0.08) (Fig. 4e), but not in TNBC (Fig. 4f). To investigate if NAC regimen is one of the driving factors affecting DRFS in TMEM doorway-high vs mid/low patients, we compared NAC treatment regimen between these two groups. There was no statistical difference in NAC treatment regimens in TMEM doorway-high and -mid/low groups (Supplementary Table 4). Even though, we observed racial disparity in macrophage density and correlation between TMEM doorway and macrophage density, there was no association between macrophage density and DRFS (Supplementary Fig. 3a). Although there was no association between DRFS and other pro-metastatic tumor markers (microvascular and CSCs density) (Supplementary Fig. 3), there was a trend towards worse DRFS in high compared to low microvascular density in TNBC (*p* = 0.06) (Supplementary Fig. 3f).

We next performed a multivariate Cox regression model and found that TMEM doorway score (high vs mid/low) is an independent prognostic indicator (HR 2.02 [95% CI 1.18–3.46], *p* = 0.01), along with stage (T3 vs T1) (HR 1.97 [95% CI 1.03–3.76], *p* = 0.04), lymph node status (positive vs negative) (HR 3.88 [95% CI 1.92–7.86], *p* = 0.0002), grade (3 vs 2) (HR 2.91 [95% CI 1.43–5.92], *p* = 0.003), and subtype (TNBC vs ER+/HER2-) (HR 1.99 [95% CI 1.1–3.58], *p* = 0.02) (Fig. 4g). Of note, we included in the multivariate analysis patients whose tumors did not fall into TN or ER+/HER2- category as “other” category because one of the goals of the study was to determine if TMEM doorway density is an independent prognostic indicator of distant recurrence regardless of cancer subtype. Multivariate Cox regression analysis among patients with ER+/HER2- breast cancer showed that there is a positive association between DRFS and TMEM doorway score (high vs mid/low) with a slightly stronger magnitude (HR 2.38 [95% CI 0.96–5.95]) but with borderline significance (*p* = 0.06) compared to entire cohort analysis. Grade (3 vs 2) remained associated with DRFS in ER+/HER2- disease (HR 3.66 [95% CI 1.45–9.27], *p* = 0.006) upon cancer subtype stratification. Although the type of surgery (mastectomy vs breast conserving therapy) was not significantly associated with DRFS in entire cohort, it was associated with DRFS in ER+/HER2- disease (HR 0.35 [95% CI 0.13–0.95], *p* = 0.04) (Supplementary Fig. 4).

These data demonstrate that out of the four pro-metastatic parameters evaluated here, only TMEM doorway score was prognostic for DRFS in patients with residual disease post-NAC.

## Discussion

**Fig. 5:**
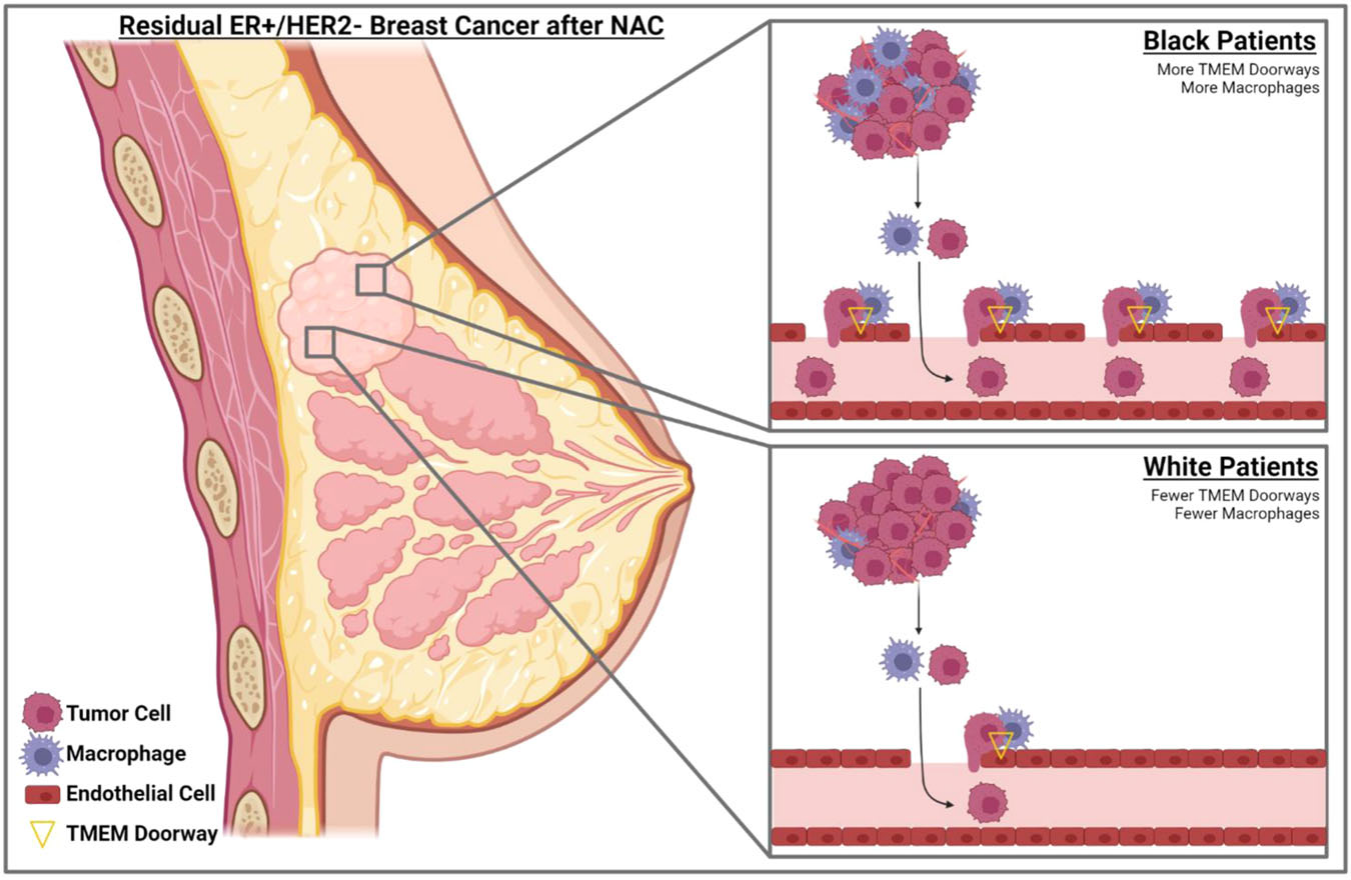
Summary of racial disparity in pro-metastatic TME of residual ER+/HER2- breast cancer after neoadjuvant chemotherapy (NAC). Black patients with residual ER+/HER2- breast cancer after NAC have higher numbers of TMEM doorways and macrophages in their tumor microenvironment compared to white patients. TMEM doorways are localized, transient vascular openings formed by Mena expressing tumor cell, perivascular macrophage, and endothelial cell. Increased permeability around TMEM doorways helps cancer cells to intravasate and disseminate to other organs. Higher TMEM doorway score can be one of the mechanisms of inferior outcome and distant recurrence free survival in Black vs white patients with residual ER+/HER2- breast cancer. This original clipart was created with BioRender.com.

In this prospective-retrospective, multi-institutional study we investigated disparity in four pro-metastatic TME parameters in residual disease post-NAC according to breast cancer subtype (ER+/HER- vs TNBC) and race (Black vs white patients), as well as the association of high density of these parameters with DRFS in all patients. We found a higher density of TMEM doorways, macrophages, and CSCs in TNBC compared to ER+/HER2-. Furthermore, we found a higher density of TMEM doorways and macrophages in residual tumors from Black, compared to white, patients with an ER+/HER2- subtype, but not TNBC. We also showed that high TMEM doorway density in residual ER+/HER2-, but not TNBC, is associated with DRFS. Taken together, these findings show that a pro-metastatic response to chemotherapy depends on cancer subtype and race and is most unfavorable in Black patients with residual ER+/HER2- disease (Fig. 5).

Over the last two decades many controlled clinical trials have demonstrated an association between Black patients and distant recurrence and several have indicated that racial disparity is more prominent in patients with ER+/HER2- disease. For example, in a randomized clinical trial including 4817 breast cancer patients, Black patients were strongly associated with inferior DRFS and overall survival in ER+/HER2- disease^9^. Likewise, in a single institution cohort of 3890 patients with invasive breast cancer, Black patients with ER+/HER2- disease had 2-fold increase in distant recurrence compared to white patients^31^. Moreover, the ECOG-ACRIN-5103 clinical trial of 4994 patients showed that Black patients with ER+/HER2- disease had worse disease free survival than white patients^10^. It needs to be emphasized that these three clinical trials have not found a racial disparity in distant recurrence in TNBC^9,10,31^. The recent NCI-sponsored trial, TAILORx, focused on ER+/HER2- disease alone, reported that Black patients were associated with a 1.60-fold increase in distant recurrence rate and an inferior overall survival^8^. Additionally, Albain et al. showed that Black patients with hormone-dependent cancers, such as pre- and post-menopausal ER+ disease, have worse outcome compared to white patients^7^. Furthermore, our group recently reported that racial disparities in DRFS for Black patients with residual ER+/HER2- but TNBC disease after NAC in a review of 8 NSABP trials^30^. However, in the present study, we only observed a trend towards worse DRFS in Black compared to white patients with residual ER+/HER2- disease. Most likely the difference in this current study did not attain statistical significance due to the tenfold smaller cohort size compared to the one by Kim et al.^30^. Consistent with the existing literature, we have not found in this current study a discrepancy in survival between Black and white patients with TNBC.

Among all breast cancer subtypes, TNBC has the poorest outcome due to a high risk of relapse, early recurrence with visceral organ metastases, and a lack of targeted therapies^32–34^. Furthermore, the aggressive nature of TNBC may mask other biological factors that would otherwise contribute to disparity in DRFS between Black and white patients. Indeed, in this current study we found that TMEM doorway score, as well as density of macrophages and CSCs, were higher in TNBC compared to ER+/HER2- disease, consistent with the higher metastatic potential associated with TNBC.

It has been shown that NAC induces repair signals in the tumor, leading to a recruitment of myeloid cells (particularly TIE2+ macrophages) into the TME^13,14^ and a subsequent increase in TMEM doorway score^15,16^. TIE2+ macrophages are crucial components of TMEM doorways, as they induce transient localized vascular openings via their secretion of vascular endothelial growth factor-A (VEGFA)^17^. Once TMEM doorways are open, MenaINV, an isoform of Mena (Mammalian Enabled, Actin regulatory protein) expressing tumor cells can intravasate^19,35,36^ and disseminate to other organs. Knowledge of this biology has important clinical implications since it has recently been demonstrated that TMEM doorway related vascular opening can be observed in patients in real time using a novel MRI approach^37^, and can be blocked by small molecule inhibitor of TIE2^38^.

Several studies have reported a correlation between high macrophage density in the breast TME and worse prognosis^39,40^. Likewise, microvascular density has been shown to correlate with inferior survival and metastasis in patients with invasive breast carcinoma^41^. In the context of racial disparity, Martin et al. showed that Black patients with breast cancer have a higher macrophage and microvasculature density in the TME compared to white patients^42^. Although we also found a greater number of macrophages in Black, compared to white, patients, we did not observe racial disparity in microvascular density. Unlike in prior reports, neither macrophage nor microvascular density were associated with DRFS in our cohort.

It is currently unclear in which clinical scenarios CSC density correlates with outcome. Some reports clearly indicate an association with worse outcome, while others do not. For example, in 48 patients with stage IV breast cancer, a high percentage of circulating CSCs was associated with inferior treatment response, survival, and progression-free survival^27^. Similarly, a meta-analysis that included 12 studies showed an association between CSCs and inferior overall survival in breast cancer^26^. However, other studies have found that CSCs and clinical outcome do not correlate^28^, which accords with the results we report here. The lack of correlation we observed between TMEM doorway density and CSCs is an unexpected finding given the recently reported finding of a positive correlation between TMEM doorway score and a high percentage of CSCs^24^. The reasons for these discrepancies need to be further investigated.

The strengths of the current study are the inclusion of patients of different racial background (Black and white patients) from several large academic institutions with similar distribution of age, time to distant recurrence status, tumor stage, and lymph node status. Although the patients were treated at different institutions, their treatments were relatively uniform (Supplementary Tables 1, 2). Of note, the criteria for calling a case ER+ changed from 10% to 1% in 2010^43^. However, about 97% of patients with ER+ disease have more than 10% of cancer cells positive for ER^44^. Therefore, subtyping cancer into ER+ category has not changed for most patients. Furthermore, the standard of care did not substantially change in the course of this study that included patients with HER2-negative disease. Anthracycline-taxane neoadjuvant chemotherapy was used in about 85% of patients, and those with ER-positive disease received adjuvant endocrine therapy for up to 10 years. An additional strength is the use of a multivariate Cox regression model that adjusts for potential confounders (Fig. 4 and Supplementary Fig. 4). Limitations are the relatively small cohort size, unknown treatment status in 10 Black and 4 white patients, and unclear effect of comorbidities (e.g., hypertension) on pro-metastatic parameters. As discussed in the results, relatively small cohort size might have underpowered our DRFS analysis and multivariate Cox regression model, especially considering nearly significant p values in ER+/HER2- group (Supplementary Fig 4). To overcome this limitation, future studies need to be designed with larger cohort size. Although the treatment status of 10 Black and 4 white patients was unknown, it is unlikely that this affected the results because for the vast majority of patients in the cohort (169) treatment regimen was known and no statistical difference in treatment status was found. Comorbidities could be one of the variates affecting survival of the patients in the study. We included potential cofounders in our multivariate Cox regression model from available patient data, however, comorbidities such as BMI or hypertension, when available, need to be taken into consideration in future studies. Additionally, knowing the adjuvant treatment such as additional endocrine therapy, radiation and chemotherapy after surgery could be affecting DRFS and would be help understand the data presented here. Unfortunately, the current sample is limited in size to control for too many potential confounders in the multivariate model. Further, one important limitation of this study is the absence of pre-chemotherapy tissue samples to observe the chemotherapy effect on TME for each patient individually. Now, we are focusing on new studies to validate our findings in larger cohorts and more detailed covariates (including comorbidities, adjuvant, endocrine, and radiation therapy regiments, etc.) with matched pre/post-chemotherapy tissue samples from patients with ER+/HER2- disease. This would guide us to investigate the impact of each limitation on our current findings and to understand the mechanism of the racial disparity in TME even further. Based on our previous work on inferior DRFS in Black patient with residual ER+/HER2- breast cancer, compared to white patients^30^, we only included Black and white patients in current study to investigate the biology of the racial disparity in these patient groups. Patients with other or unknown races were excluded in this study. To expand racial disparity, new studies could be designed including other races and/or ethnicities.

In conclusion, this study demonstrates the existence of a racial disparity in pro-metastatic TME in Black, compared to white, women with residual ER+/HER2- disease after NAC. This work provides a foundation for the development of companion diagnostics such as TMEM-MRI activity^37^ to facilitate the evaluation of systemic therapies on the metastatic dissemination, and therapies to block TMEM doorway activity^38^, which may ultimately contribute to reducing racial disparity in breast cancer.

## Methods

### Study design

This was a prospective-retrospective, multi-institutional case-control study performed in accordance with REMARK guidelines^45,46^. Patients were selected from four participating New York Pathology Oncology Group (NYPOG-https://einsteinmed.edu/research/groups/ny-pathology-oncology/) institutions (Montefiore Medical Center; New York-Presbyterian/Weill Cornell Medical Center; NYU Langone Health; Memorial Sloan Kettering Cancer Center). The study was approved by the Institutional Review Board of each institution. The written consent from patients was not obtained because we only used leftover archival tissue that was excised for standard clinical care for diagnostic or therapeutic purposes years before the initiation of this study. Due to the timing of the study many subjects are deceased. Female patients over age of 18, diagnosed with invasive ductal carcinoma (IDC) between 2004 and 2014, who received neoadjuvant chemotherapy and had sufficient residual cancer for staining (defined as >5 mm) were included. Patients with a prior personal history of breast or other cancers, bilateral cancers, and distant metastatic disease at the time of presentation were excluded from the study (Fig. 1). Cases were defined as patients with localized breast cancer treated with NAC who subsequently developed distant recurrence, whereas controls were defined as patients who did not develop distant recurrence. Detailed NAC regimens can be found in Supplementary Tables 1, 2.

Although it is now understood that race is a social construct, race was self-identified per the patient’s electronic medical records. Only patients of Black or white patients were included. Patients with ‘Other’ or unknown races were excluded from the study. Distant recurrence was defined as clinical or radiographic evidence of disease recurrence outside of the breast, chest wall, and axillary lymph nodes, with or without tissue confirmation. Primary endpoints were TMEM doorway score; percentage of tumor cells expressing high levels of SOX9, which indicates activation of stem program^24^; and time to distant recurrence, measured in months from date of diagnosis. Secondary endpoints were macrophage and microvascular density.

### Cohort assembly and tissue collection

The tumor registry at each institution was queried and all cases were reviewed by a clinician to ensure that inclusion and exclusion criteria were met. A cohort of 216 patients who met the inclusion and exclusion criteria were assembled from the NYPOG institutions. The representative Formalin Fixed Paraffin Embedded (FFPE) blocks created at the time of surgical excision and stored in pathology archives at each institution were selected and five sequential sections were cut. Of the cohort 216 patients, 33 were excluded due to poor tissue quality, as determined by pathologists (MO, SA).

### Slide staining & scanning

Of the five sequential sections, one was stained for TMEM doorways, and the other for the stem cell marker (SOX9) and DAPI (to mark nuclei).

TMEM doorways were visualized by triple immunostaining as per a previously validated protocol^22,23^ identifying Mena-overexpressing tumor cells, macrophages, and endothelial cells. Cancer stem cells were identified by immunofluorescent staining of SOX9 (anti-rabbit Millipore 3205915, 1:100 dilution) and DAPI (1:1000 dilution) on an adjacent sequential section. Alexa Fluor-546 goat anti-rabbit (H + L) (Thermofisher, Cat# A11035, 1:200 dilution) was used as a secondary antibody. The slides were digitally scanned at 20x on a 3D Histech P250 High-Capacity Slide Scanner.

### TMEM doorway quantification

The digitally scanned slides were first imported into the Visiopharm image analysis software, Vis (Visiopharm, Hørsholm, Denmark). Next, in Vis, pathologists (MO, SA) identified approximately ten high power (20X) microscope fields best suitable for analysis based upon appropriate pathological criteria (e.g., lack of tumor necrosis, inflammation, tissue folds). Each 20X field covers a 660 × 880 µm^2^ area, which is 4 times the area of a 40X field previously used for TMEM doorway quantification^23,47,48^. Next, TMEM doorways were quantified using previously published algorithms^47^ in all the 20X microscope fields and the sum of TMEM doorways in these fields, divided by a conversion factor of 4, was used as the TMEM doorway score for the patient sample, which is consistent with previously published methods^23,47,48^. TMEM doorway-high patients were defined as those in the highest tertile of TMEM doorway score.

### Quantification of macrophage and microvascular densities

In the original TMEM doorway quantification algorithm^47^, tumor, macrophage, and vessel areas were identified across entire region of interest (ROI). Macrophage area divided by the ROI area was used to determine the macrophage density. Likewise, vessel area divided by the ROI area determined the vascular density.

### Nuclear SOX9 image analysis

We analyzed the percentage of nuclear SOX9 cells in Vis. We used the same ten, high-powered fields (20X) that were used for TMEM doorway quantification. The ROIs were captured using the screen capture tool and exported as bitmap (BMP) image files (lossless and uncompressed), allowing for accurate quantification of immunofluorescence intensities. Using the image analysis package, Fiji^49^, each image was split into separate color channels and the red (SOX9) and blue (nuclei) channels were used for further analysis. Again in Fiji, a machine learning algorithm, implemented via the Trainable Weka Segmentation plugin^50^ was used to segment the nuclei (blue) channel. The Trainable Weka Segmentation plugin was again run with separate training on the SOX9 (red) channel to segment the tumor regions. SOX9 mean fluorescence intensity measurements were made for nuclei in the tumor regions. Nuclear SOX9 intensity was quantified in each nucleus, and SOX9^high^ cells above a specified intensity threshold were counted. It has been determined that CSCs constitute no more than 5% of cancer cells in untreated tumors^24^. Thus, the threshold for detecting CSCs was determined using cases expected to have the lowest CSC density - untreated ER+/HER2- breast cancers from white patients. We showed previously that CSC density correlates with TMEM doorway density^24^. In addition, we showed here that ER+/HER2- cancers have lower TMEM doorway density than TNBC and that breast cancers from white patients have lower TMEM doorway density than cancers from Black patients. Furthermore, TMEM doorway density may increase as a response to chemotherapy^15^. Hence the threshold was calculated using three different untreated ER+/HER2- cancers from white patients and the cutoffs averaged. This intensity threshold was then applied to all samples. SOX9^high^ cell numbers were aggregated from all ten high-powered fields to get a single percentage of nuclear SOX9^high^ cells number for each patient.

### Sample size and power calculation

For each race group with at least *N* = 50 non-recurrences and *N* = 25 recurrences, with a two-sided type I error rate of not more than 5%, the study has 80% power to detect a difference of 0.61 SD in each marker between recurrences and non-recurrences. Thus, our study has adequate power to detect a moderate level of effect size.

### Statistical analysis

Patient and tumor characteristics, including age (years); surgery type (breast conserving therapy vs mastectomy); post-NAC tumor size (> 5 cm, 2–5 cm, < 2 cm); post-NAC nodal status (positive vs. negative); Nottingham histologic grade (1, 2, 3); tumor subtype (TNBC vs ER+/HER2-); and tumor markers, including TMEM doorway score; density of SOX9^High^ cancer cells; macrophage density; and microvascular density were compared between Black and white patients using Wilcoxon rank sum tests for continuous variables and chi-squared tests or Fisher’s exact tests for categorical variables. Spearman correlation was calculated between each pair of tumor markers.

The primary outcome variable was DRFS, defined as time from diagnosis to first distant relapse or a second primary cancer. Death prior to a distant recurrence or second primary cancer was censored. Kaplan-Meier survival curves and log-rank tests were used to compare DRFS between racial groups and between categorized pro-metastatic tumor characteristics (e.g., high vs low/mid tertile). A multivariate Cox proportional hazard model was used to examine the association between TMEM doorway score and DRFS, adjusting for race, age, surgery type, tumor size, nodal status, tumor grade, tumor subtype. The proportionality of the Cox model was examined using Schoenfeld residuals^51,52^.

Statistical significance was specified a priori as *p* < 0.05 and the two-side p-values were reported. All analyses were conducted using SAS 9.4 (SAS Institute Inc., Cary, NC 2014) and GraphPad Prism version 9.1 (Dotmatics, Boston, MA).

### Reporting summary

Further information on research design is available in the Nature Research Reporting Summary linked to this article.

## Supplementary information

Supplementary Materials

Reporting Summary

## Data Availability

The data generated in this study are available upon request from the corresponding author.
