# Supplementary material for: Racial disparity in tumor microenvironment and distant recurrence in residual breast cancer after neoadjuvant chemotherapy
Source: NPJ Breast Cancer. 2023 Jun 13;9:52. doi: 10.1038/s41523-023-00547-w (PMC10264351; doi:10.1038/s41523-023-00547-w)
Supplement: Supplementary file 1 — Supplementary Materials [file 41523_2023_547_MOESM1_ESM.pdf]

Supplementary Figure 1.

a TMEM Doorway-high

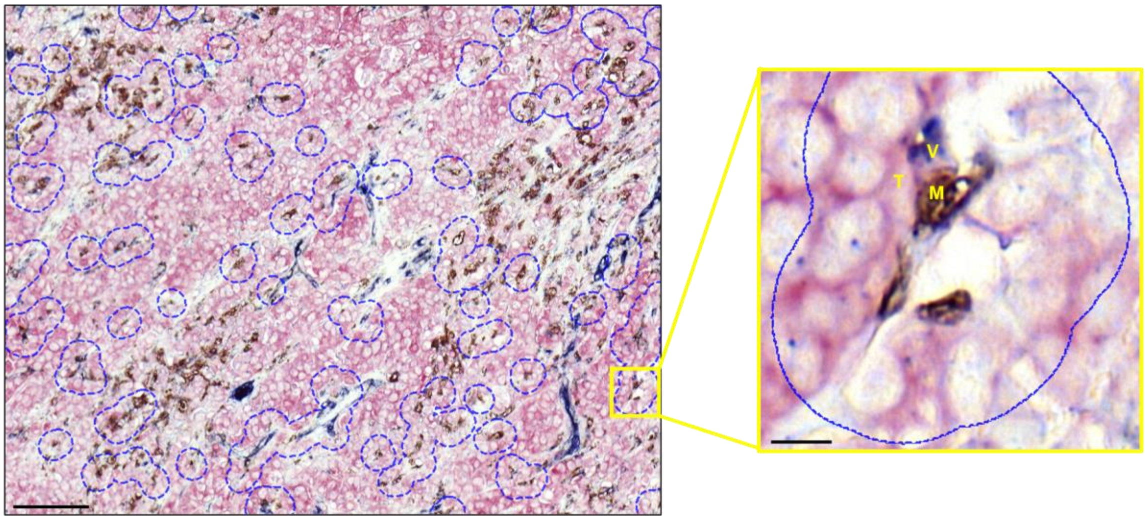

b TMEM Doorway-mid/low

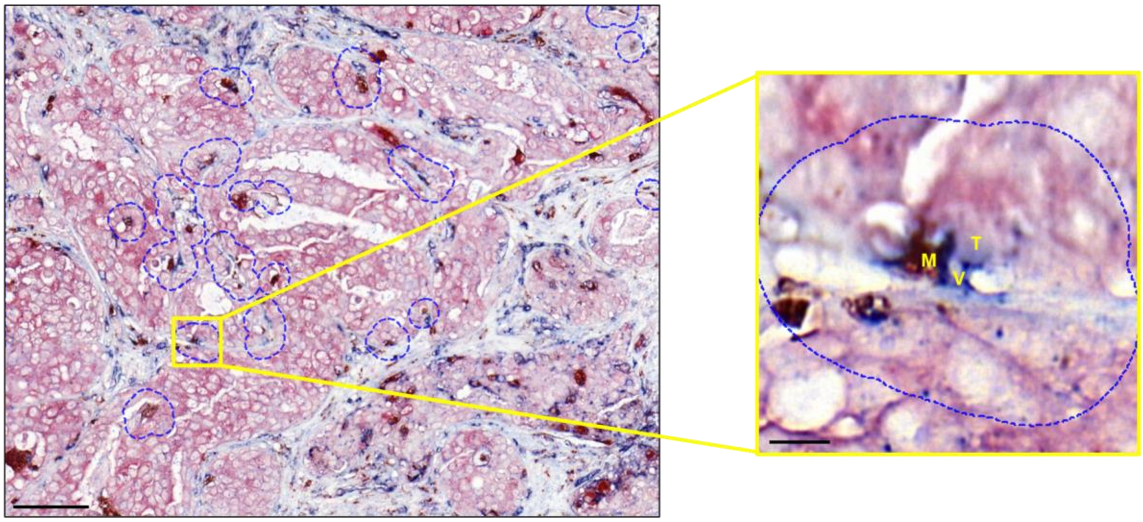

Supplementary Figure 1. Representative images for TMEM Doorway-high (a) and TMEM Doorway-mid/low (b). Tumor cells are stained for PanMena (pink), CD68 macrophages (brown), CD31 endothelial cells, representing vasculature (blue). Blue-dashed circles show TMEM doorways, drawn by automated TMEM doorway analysis. T: Tumor Cell, M: Macrophage, V: Vasculature. Scale bar on the lower power images (left), 100 µm. Scale bar on the higher power images (right), 10 µm.

Supplementary Figure 2.

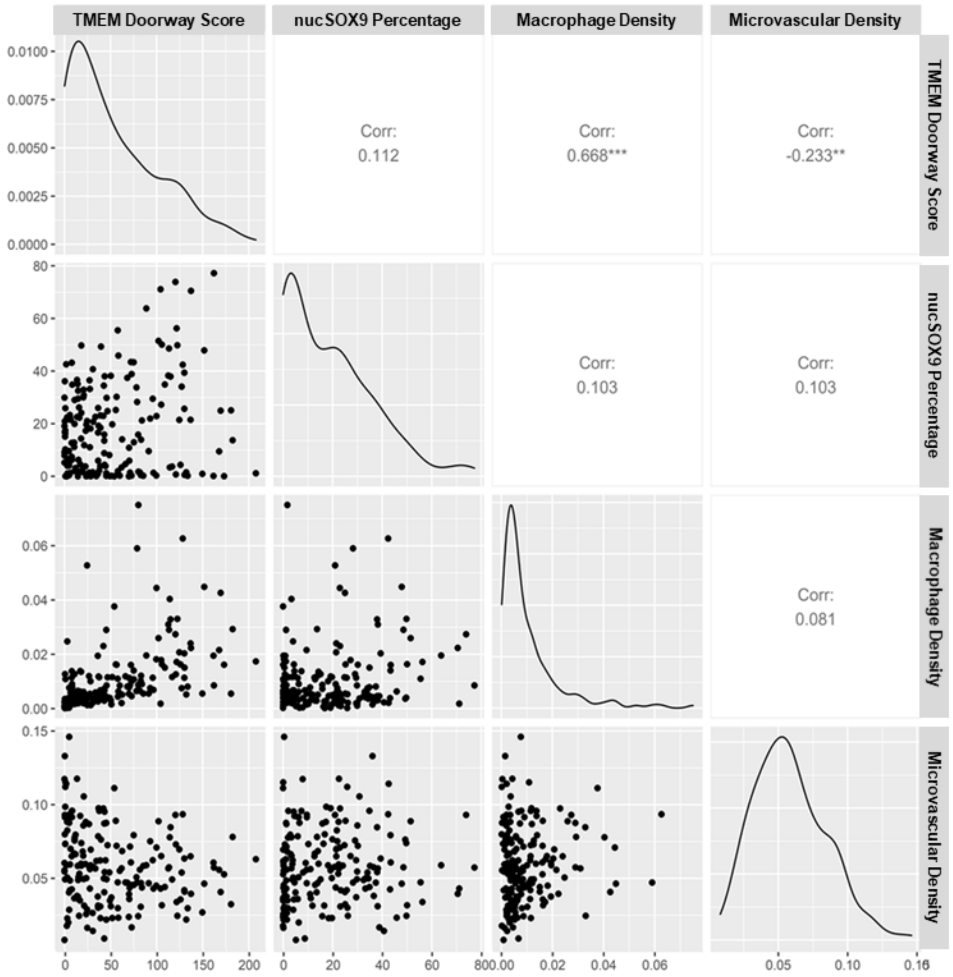

Supplementary Figure 2. Correlation between all pro-metastatic TME parameters, entire cohort. Spearman correlation analysis showed a positive correlation between TMEM doorway score and macrophage density (correlation coefficient= 0.67). Diagonally placed curve graphs represent distribution of the parameters.

Supplementary Figure 3.

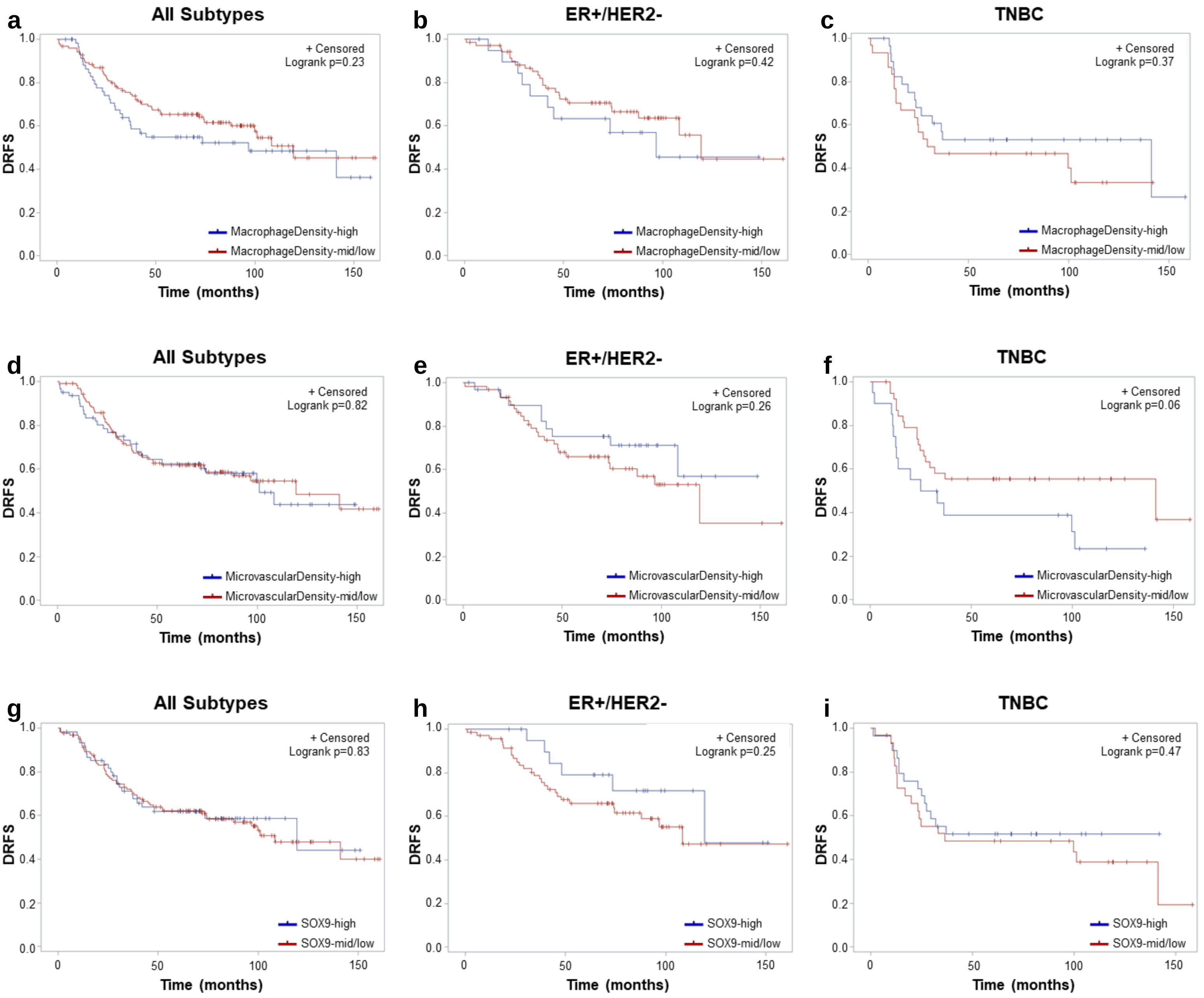

Supplementary Figure 3. There is no association between DRFS and pro-metastatic tumor markers; entire cohort, ER+/HER2-, and TNBC. a-c, DRFS in Macrophage Density-high vs -mid/low patients; entire cohort (a, p=0.23), ER+/HER2- (b, p=0.42), and TNBC (c, p=0.37). d-f, DRFS in Microvascular Density-high vs -mid/low patients; entire cohort (d, p=0.82), ER+/HER2- (e, p=0.26), and TNBC (f, p=0.06). g-i, DRFS in SOX9-high vs -mid/low patients; entire cohort (g, p=0.83), ER+/HER2- (h, p=0.25), and TNBC (i, p=0.47). Kaplan-Meier survival curves and log-rank tests are used for DRFS analysis (a-i). DRFS: Distant Recurrence Free Survival, ER+: Estrogen Receptor positive, TNBC: Triple Negative Breast Cancer, nucSOX9: nuclear SOX9.

Supplementary Figure 4.

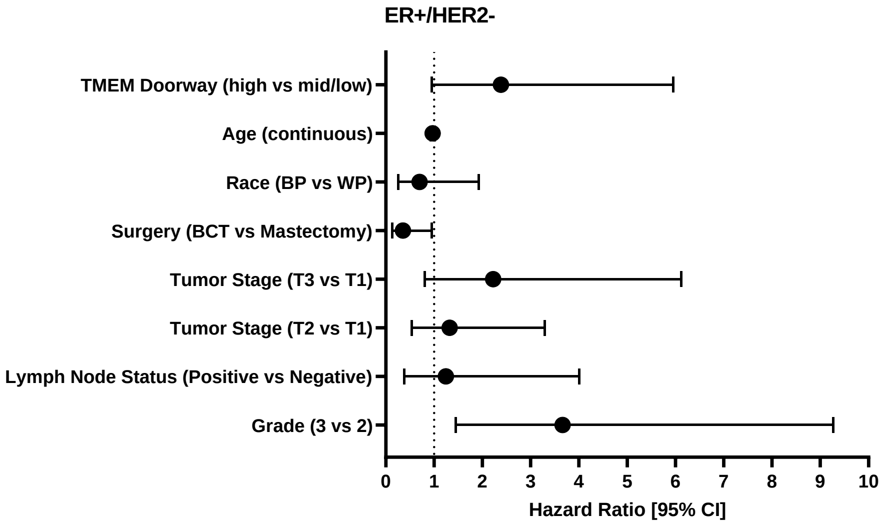

HR [95% CI]	P Value
2.38 [0.96 – 5.95]	0.06
0.97 [0.93 – 1.01]	0.12
0.70 [0.25 – 1.93]	0.49
0.35 [0.13 – 0.95]	0.04
2.22 [0.81 – 6.12]	0.12
1.32 [0.53 – 3.29]	0.55
1.24 [0.39 – 4.01]	0.72
3.66 [1.45 – 9.27]	0.006

Supplementary Figure 4. Cox regression model for covariates in ER+/HER2- group shows trend towards TMEM doorway as an independent tumor marker. Error bars represent 95% confidence intervals. Two-sided p values are reported. n=86, patients with unknown status, n=5, are excluded.

Supplementary Table 1. Neoadjuvant Chemotherapy Regimen, Entire Cohort

	Number of Patients (%)			
	All Patients N=183 (100)	Black Patients N=96 (52.5)	White Patients N=87 (47.5)	P Value
**Neoadjuvant Chemotherapy (NAC)**				0.33
Taxane-containing	157 (85.8)	80 (83.3)	77 (88.5)	
ACT	132 (84.1)	70 (87.5)	62 (80.5)	0.12
AT	3 (1.9)	2 (2.5)	1 (1.3)	
CT	5 (3.2)	0 (0)	5 (6.5)	
T	17 (10.8)	8 (10)	9 (11.7)	
No taxane	12 (6.6)	6 (6.3)	6 (6.9)	
Unknown[a]	14 (7.7)	10 (10.4)	4 (4.6)	
**All Treatments**				0.51
NAC	140 (76.5)	72 (75)	68 (78.2)	
NAC + Endocrine	2 (1.1)	1 (1.0)	1 (1.1)	
NAC + HER2 inhibitor	23 (12.6)	12 (12.5)	11 (12.6)	
NAC + Radiotherapy	4 (2.2)	1 (1.0)	3 (3.4)	
Unknown[a]	14 (7.7)	10 (10.4)	4 (4.6)	

Supplementary Table 1. Neoadjuvant chemotherapy regimen, entire cohort. Chi-squared test is used for the statistical analysis. [a]Detailed neoadjuvant chemotherapy information is not available. A: anthracycline, C: cyclophosphamide, T: taxane.

Supplementary Table 2. Neoadjuvant Chemotherapy Regimen by Subtype

	ER+/HER2- Number of Patients (%)				TNBC Number of Patients (%)			
	All Patients N=91 (100)	Black Patients N=41 (45.1)	White Patients N=50 (54.9)	P Value	All Patients N=59 (100)	Black Patients N=37 (62.7)	White Patients N=22 (37.3)	P Value
**NAC**				0.15				0.38
Taxane-containing	78 (85.7)	32 (78.1)	46 (92)		53 (89.8)	32 (86.5)	21 (95.5)	
ACT	66 (84.6)	27 (84.4)	39 (84.8)	0.63	50 (94.3)	32 (100)	18 (85.7)	0.09
AT	2 (2.6)	1 (3.1)	1 (2.2)		0 (0)	0 (0)	0 (0)	
CT	2 (2.6)	0 (0)	2 (4.3)		2 (3.8)	0 (0)	2 (9.5)	
T	8 (10.3)	4 (12.5)	4 (8.7)		1 (1.9)	0 (0)	1 (4.8)	
No taxane	5 (5.5)	3 (7.3)	2 (4)		3 (5.1)	2 (5.4)	1 (4.5)	
Unknown[a]	8 (8.8)	6 (14.6)	2 (4)		3 (5.1)	3 (8.1)	0 (0)	
**All Treatments**				0.29				0.17
NAC	77 (84.6)	33 (80.5)	44 (88)		56 (94.9)	34 (91.9)	22 (100)	
NAC + Endocrine	2 (2.2)	1 (2.4)	1 (2)		0 (0)	0 (0)	0 (0)	
NAC + HER2 inhibitor	0 (0)	0 (0)	0 (0)		0 (0)	0 (0)	0 (0)	
NAC + Radiotherapy	4 (4.4)	1 (2.4)	3 (6)		0 (0)	0 (0)	0 (0)	
Unknown[a]	8 (8.8)	6 (14.6)	2 (4)		3 (5.1)	3 (8.1)	0 (0)	

Supplementary Table 2. Neoadjuvant chemotherapy regimen by subtype. Chi-squared test is used for the statistical analysis. [a]Detailed neoadjuvant chemotherapy information is not available. A: anthracycline, C: cyclophosphamide, T: taxane, ER+: estrogen receptor positive, TNBC: triple negative breast cancer.

Supplementary Table 3. Patient Characteristics by Subtype

	ER+/HER2- Number of Patients (%)				TNBC Number of Patients (%)			
	All Patients N=91 (100)	Black Patients N=41 (45.1)	White Patients N=50 (54.9)	P Value	All Patients N=59 (100)	Black Patients N=37 (62.7)	White Patients N=22 (37.3)	P Value
Distant Recurrence				0.11				1
Yes	33 (36.3)	19 (46.3)	14 (28)		32 (54.2)	20 (54.1)	12 (54.5)	
No	58 (63.7)	22 (53.7)	36 (72)		27 (45.8)	17 (45.9)	10 (45.5)	
Age				0.13				0.44
Mean [SD]	52 [12.4]	49.6 [11.3]	54 [12.9]		52.5 [9.7]	53.4 [8.3]	50.9 [11.8]	
Median [IQR]	50 [43.5,61]	49 [42,56]	52 [44,62]		52 [47,58]	52 [48,58]	53.5[42.5,57]	
Range	29 – 95	29 – 78	31 – 95		30 - 75	33 - 70	30 - 75	
Time to Distant Recurrence, Months				0.78				0.14
Mean [SD]	67.1 [36.9]	66.5 [38.2]	67.7 [36.1]		55.4 [44.5]	62.3 [47.9]	43.6 [36.1]	
Median [IQR]	71.5 [35.7,94.7]	68.6 [33,93.4]	72.7[42.2,95.3]		36.6[15.1,95.4]	37.1[16.3,99.7]	31.3[13,63.5]	
Range	1 – 160.7	12 – 151	1 – 160.7		1.2 – 158.2	10.6 – 158.2	1.2 – 113.5	
Surgery				0.004				0.55
Mastectomy	60 (65.9)	34 (82.9)	26 (52)		36 (61)	21 (56.8)	15 (68.2)	
BCT	31 (34.1)	7 (17.1)	24 (48)		23 (39)	16 (43.2)	7 (31.8)	
Tumor Stage (ypT)				0.91				0.49
T1 (<2cm)	38 (41.8)	18 (43.9)	20 (40)		20 (33.9)	14 (37.8)	6 (27.3)	
T2 (2-5 cm)	40 (44)	17 (41.5)	23 (46)		25 (42.4)	16 (43.2)	9 (40.9)	
T3 (>5 cm)	13 (14.3)	6 (14.6)	7 (14)		14 (23.7)	7 (18.9)	7 (31.8)	
Lymph Node Status (ypN)				0.01				0.52
Positive	70 (76.9)	37 (90.2)	33 (66)		33 (55.9)	19 (51.4)	14 (63.6)	
Negative	21 (23.1)	4 (9.8)	17 (34)		26 (44.1)	18 (48.6)	8 (36.4)	
Grade				0.01				0.4
1	4 (4.4)	0 (0)	4 (8)		0 (0)	0 (0)	0 (0)	
2	40 (44)	15 (36.6)	25 (50)		6 (10.2)	5 (13.5)	1 (4.5)	
3	42 (46.2)	26 (63.4)	16 (32)		51 (86.4)	31 (83.8)	20 (90.9)	
Unknown	5 (5.5)	0 (0)	5 (10)		2 (3.4)	1 (2.7)	1 (4.5)	

Supplementary Table 3. Patient characteristics by subtype. Wilcoxon rank sum test is used for continuous variables. Chi-squared tests or Fisher's exact tests are used for categorical variables. SD: standard deviation, IQR: interquartile range, BCT: breast conserving therapy, yPT: tumor stage after neoadjuvant chemotherapy, yPN: lymph node status after neoadjuvant chemotherapy, ER+: estrogen receptor positive, TNBC: triple negative breast cancer.

Supplementary Table 4. Neoadjuvant Chemotherapy Regimen by TMEM Doorway Score

	Number of Patients (%)			
	All Patients N=183 (100)	TMEM Doorway-high N=61 (33.7)	TMEM doorway-mid/low N=122 (66.7)	P Value
**Neoadjuvant Chemotherapy (NAC)**				0.73
Taxane-containing	157 (85.8)	51 (83.6)	106 (86.9)	
ACT	132 (84.1)	42 (82.4)	90 (84.9)	0.61
AT	3 (1.9)	2 (3.9)	1 (0.9)	
CT	5 (3.2)	2 (3.9)	3 (2.8)	
T	17 (10.8)	5 (9.8)	12 (11.3)	
No taxane	12 (6.6)	4 (6.6)	8 (6.6)	
Unknown[a]	14 (7.7)	6 (9.8)	8 (6.6)	
**All Treatments**				0.83
NAC	140 (76.5)	47 (77.1)	93 (76.2)	
NAC + Endocrine	2 (1.1)	1 (1.6)	1 (0.8)	
NAC + HER2 inhibitor	23 (12.6)	6 (9.8)	17 (13.9)	
NAC + Radiotherapy	4 (2.2)	1 (1.6)	3 (2.5)	
Unknown[a]	14 (7.7)	6 (9.8)	8 (6.6)	

Supplementary Table 4. Neoadjuvant chemotherapy regimen by TMEM doorway score. Chi-squared test is used for the statistical analysis. [a]Detailed neoadjuvant chemotherapy information is not available. A: anthracycline, C: cyclophosphamide, T: taxane.
