# Supplementary material for: Racial disparity in tumor microenvironment and distant recurrence in residual breast cancer after neoadjuvant chemotherapy
Source: NPJ Breast Cancer. 2023 Jun 13;9:52. doi: 10.1038/s41523-023-00547-w (PMC10264351; doi:10.1038/s41523-023-00547-w)
Supplement: Supplementary file 2 — Reporting Summary [file 41523_2023_547_MOESM2_ESM.pdf]

nature portfolio

Corresponding author(s): Maja H. Oktay - NPJBCANCER-02372

Last updated by author(s): Apr 25, 2023

Reporting Summary

Nature Portfolio wishes to improve the reproducibility of the work that we publish. This form provides structure for consistency and transparency in reporting. For further information on Nature Portfolio policies, see our Editorial Policies and the Editorial Policy Checklist.

Statistics

For all statistical analyses, confirm that the following items are present in the figure legend, table legend, main text, or Methods section.

n/a	Confirmed	
☐	☒	The exact sample size (*n*) for each experimental group/condition, given as a discrete number and unit of measurement
☒	☐	A statement on whether measurements were taken from distinct samples or whether the same sample was measured repeatedly
☐	☒	The statistical test(s) used AND whether they are one- or two-sided<br>*Only common tests should be described solely by name; describe more complex techniques in the Methods section.*
☐	☒	A description of all covariates tested
☐	☒	A description of any assumptions or corrections, such as tests of normality and adjustment for multiple comparisons
☐	☒	A full description of the statistical parameters including central tendency (e.g. means) or other basic estimates (e.g. regression coefficient) AND variation (e.g. standard deviation) or associated estimates of uncertainty (e.g. confidence intervals)
☐	☒	For null hypothesis testing, the test statistic (e.g. *F*, *t*, *r*) with confidence intervals, effect sizes, degrees of freedom and *P* value noted<br>*Give P values as exact values whenever suitable.*
☒	☐	For Bayesian analysis, information on the choice of priors and Markov chain Monte Carlo settings
☒	☐	For hierarchical and complex designs, identification of the appropriate level for tests and full reporting of outcomes
☐	☒	Estimates of effect sizes (e.g. Cohen's *d*, Pearson's *r*), indicating how they were calculated

Our web collection on statistics for biologists contains articles on many of the points above.

Software and code

Policy information about availability of computer code

Data collection	No software was used for data collection.
Data analysis	Custom made code were used in TMEM doorway quantification. Details of this analysis were described in the following study: doi.org/10.3390/cancers12040846. Nuclear SOX9 was analyzed by using Fiji (doi.org/10.1038/nmeth.2019) and Trainable Weka Segmentation plugin (doi.org/10.1093/bioinformatics/btx180).

For manuscripts utilizing custom algorithms or software that are central to the research but not yet described in published literature, software must be made available to editors and reviewers. We strongly encourage code deposition in a community repository (e.g. GitHub). See the Nature Portfolio guidelines for submitting code & software for further information.

Data

Policy information about availability of data

All manuscripts must include a data availability statement. This statement should provide the following information, where applicable:

- Accession codes, unique identifiers, or web links for publicly available datasets
- A description of any restrictions on data availability
- For clinical datasets or third party data, please ensure that the statement adheres to our policy

The data generated in this study are available upon request from the corresponding author.

Human research participants

Policy information about studies involving human research participants and Sex and Gender in Research.

Reporting on sex and gender	*Use the terms sex (biological attribute) and gender (shaped by social and cultural circumstances) carefully in order to avoid confusing both terms. Indicate if findings apply to only one sex or gender; describe whether sex and gender were considered in study design whether sex and/or gender was determined based on self-reporting or assigned and methods used. Provide in the source data disaggregated sex and gender data where this information has been collected, and consent has been obtained for sharing of individual-level data; provide overall numbers in this Reporting Summary. Please state if this information has not been collected. Report sex- and gender-based analyses where performed, justify reasons for lack of sex- and gender-based analysis.*
Population characteristics	*Describe the covariate-relevant population characteristics of the human research participants (e.g. age, genotypic information, past and current diagnosis and treatment categories). If you filled out the behavioural & social sciences study design questions and have nothing to add here, write "See above."*
Recruitment	*Describe how participants were recruited. Outline any potential self-selection bias or other biases that may be present and how these are likely to impact results.*
Ethics oversight	*Identify the organization(s) that approved the study protocol.*

Note that full information on the approval of the study protocol must also be provided in the manuscript.

Field-specific reporting

Please select the one below that is the best fit for your research. If you are not sure, read the appropriate sections before making your selection.

☒ Life sciences ☐ Behavioural & social sciences ☐ Ecological, evolutionary & environmental sciences

For a reference copy of the document with all sections, see nature.com/documents/nr-reporting-summary-flat.pdf

Life sciences study design

All studies must disclose on these points even when the disclosure is negative.

Sample size	For each race group with at least N=50 non-recurrences and N=25 recurrences, with a two-sided type I error rate of not more than 5%, the study has 80% power to detect a difference of 0.61 SD in each marker between recurrences and non-recurrences. Thus, our study has adequate power to detect a moderate level of effect size.
Data exclusions	Of the cohort 216 patients, 33 were excluded due to poor tissue quality, as determined by pathologists (MO, SA).
Replication	Replicates were not indicated.
Randomization	This was a retrospective-prospective study of archival breast cancer tissue samples. Therefore, randomization was not possible. Covariates could not be controlled, but they were included in the multivariate cox regression model based on patients' clinical history.
Blinding	Analysis of the tumor markers were done blindly.

Reporting for specific materials, systems and methods

We require information from authors about some types of materials, experimental systems and methods used in many studies. Here, indicate whether each material, system or method listed is relevant to your study. If you are not sure if a list item applies to your research, read the appropriate section before selecting a response.

Materials & experimental systems

n/a	Involved in the study	
☐	☒	Antibodies
☒	☐	Eukaryotic cell lines
☒	☐	Palaeontology and archaeology
☒	☐	Animals and other organisms
☐	☒	Clinical data
☒	☐	Dual use research of concern

Methods

n/a	Involved in the study	
☒	☐	ChIP-seq
☒	☐	Flow cytometry
☒	☐	MRI-based neuroimaging

Antibodies

Antibodies used	CD-31 antibody (clone JC70A; 1:800 dilution; DAKO, Santa Clara, CA, USA) with Bond Epitope Retrieval Solution 2 and Vector Blue chromogen, CD-68 antibody (clone PG-M1; 1:300 dilution; DAKO) with antigen retrieval using Bond Epitope Retrieval Solution 1 and 3,3'-Diaminobenzidine (DAB) chromogen, anti-pan-Mena antibody (P/N: 610692, BD Biosciences, San Jose, CA, USA) that stain all isoforms of Mena, macrophages with Fast Red chromogen (Bond Polymer Refine Red Detection, Leica Biosystems, Buffalo Grove, IL, USA), SOX9 antibody (anti-rabbit Millipore 3205915), Alexa Fluor-546 goat anti-rabbit (H+L) (Thermofisher, Cat# A11035).
Validation	All the antibodies used in this study were validated in our previously published papers: doi.org/10.3390/cancers12040846, doi.org/10.1038/s41467-021-27308-2.

Clinical data

Policy information about clinical studies

All manuscripts should comply with the ICMJE guidelines for publication of clinical research and a completed CONSORT checklist must be included with all submissions.

Clinical trial registration	*Provide the trial registration number from ClinicalTrials.gov or an equivalent agency.*
Study protocol	*Note where the full trial protocol can be accessed OR if not available, explain why.*
Data collection	*Describe the settings and locales of data collection, noting the time periods of recruitment and data collection.*
Outcomes	*Describe how you pre-defined primary and secondary outcome measures and how you assessed these measures.*
